# Optimization of CRISPR/Cas9 genome editing in cotton by improved sgRNA expression

**DOI:** 10.1186/s13007-018-0353-0

**Published:** 2018-10-03

**Authors:** Lu Long, Dan-Dan Guo, Wei Gao, Wen-Wen Yang, Li-Pan Hou, Xiao-Nan Ma, Yu-Chen Miao, Jose Ramon Botella, Chun-Peng Song

**Affiliations:** 1 State Key Laboratory of Cotton Biology, Henan Key Laboratory of Plant Stress Biology, School of Life Science, Henan University, Kaifeng, 475004 Henan People’s Republic of China; 2 School of Agriculture and Food Sciences, University of Queensland, Brisbane, QLD 4072 Australia

**Keywords:** CRISPR/Cas9, *U6* promoter, Genome editing, Target mutagenesis, Transient expression

## Abstract

**Background:**

When developing CRISPR/Cas9 systems for crops, it is crucial to invest time characterizing the genome editing efficiency of the CRISPR/Cas9 cassettes, especially if the transformation system is difficult or time-consuming. Cotton is an important crop for the production of fiber, oil, and biofuel. However, the cotton stable transformation is usually performed using *Agrobacterium tumefaciens* taking between 8 and 12 months to generate T_0_ plants. Furthermore, cotton is a heterotetraploid and targeted mutagenesis is considered to be difficult as many genes are duplicated in this complex genome. The application of CRISPR/Cas9 in cotton is severely hampered by the long and technically challenging genetic transformation process, making it imperative to maximize its efficiency.

**Results:**

In this study, we provide a new system to evaluate and validate the efficiency of CRISPR/Cas9 cassettes in cotton using a transient expression system. By using this system, we could select the most effective CRISPR/Cas9 cassettes before the stable transformation. We have also optimized the existing cotton CRISPR/Cas9 system to achieve vastly improved mutagenesis efficiency by incorporating an endogenous *GhU6* promoter that increases sgRNA expression levels over the *Arabidopsis AtU6*-*29* promoter. The 300 bp *GhU6.3* promoter was cloned and validated using the transient expression system. When sgRNAs were expressed under the control of the *GhU6.3* promoter in CRISPR/Cas9 cassettes, expression levels were 6–7 times higher than those provided by the *AtU6*-*29* promoter and CRISPR/Cas9-mediated mutation efficiency was improved 4–6 times.

**Conclusions:**

This study provides essential improvements to maximize CRISPR/Cas9-mediated mutation efficiency by reducing risk and workload for the application of CRISPR/Cas9 approaches in the targeted mutagenesis of cotton.

**Electronic supplementary material:**

The online version of this article (10.1186/s13007-018-0353-0) contains supplementary material, which is available to authorized users.

## Background

Targeted genome editing is an extremely useful tool for basic and applied plant research and a number of techniques such as meganucleases, zinc finger nucleases (ZFNs) and transcription activator-like effectors nucleases (TALENs) had been available for some time [1–3]. Nevertheless, the technical complexity of the above mentioned technologies precluded its adoption by the wider scientific community until the advent of CRISPR/Cas9 (clustered regularly interspaced short palindromic repeats/CRISPR-associated protein 9). The intrinsic versatility, simplicity and high efficiency of CRISPR/Cas9 has resulted in an explosion of research using genome-editing as the preferred method to generate precise alterations in the genome of numerous plant species [4–10]. CRISPR/Cas9 derives from a microbial adaptive immune system and its major components are the Cas9 nuclease capable of producing double strand breaks and a small guide RNA (sgRNA) which directs the Cas9 protein to the target site. A number of factors influence the efficiency of the CRISPR/Cas9 system with strong expression of Cas9 and sgRNA being essential to obtain high mutation rates [9, 10].

U6 small nuclear RNAs (snRNAs) are non-coding RNAs involved in intron splicing during the production of mature mRNA molecules in eukaryotic cells. The *U6* promoter is a class III RNA polymerase III promoter that has been frequently used to drive high expression levels of small RNAs in plants and animals [11, 12] and has been the preferred choice to drive sgRNA expression in CRISPR/Cas9 vectors [13, 14]. In addition, the *U6* promoter has a highly conserved transcription start site starting with a guanine nucleotide, which helps to improve the homogeneity of the transcribed sgRNA molecule and reduce off-target effects [12]. CRISPR/Cas9 vector systems using the *U6* promoter to drive sgRNA expression have been successfully used in various plant species, with the *OsU6a*, *OsU6b* and *OsU6c* promoters from rice being the most commonly used for monocotyledons, and the *Arabidopsis AtU6*-*1* and *AtU6*-*29* promoters being the preferred ones for dicotyledons [10, 15, 16]. It has nevertheless become clear that the use of species-specific *U6* promoters can result in increased sgRNA expression and thus enhanced editing efficiency [9, 17]. In soybean, for example, sgRNA levels driven by the endogenous *GmU6* promoter were twice higher than those obtained using the *Arabidopsis AtU6*-*26* promoter, resulting in massive improvements in gene editing efficiency (14.7–20.2% for *GmU6* vs. 3.2–9.7% for *AtU6*-*26*) [17]. It is also important to keep in mind that plant genomes contain multiple *U6* genes with different expression levels with the corollary that not all *U6* promoters are equally efficient in driving gene expression [18, 19]. When developing CRISPR/Cas9 systems for new species it is important to invest time characterizing exogenous and endogenous *U6* promoters to choose the optimal one, especially if the transformation system is difficult or time consuming.

Cotton is an important crop for the production of fiber, oil and biofuel. The genome of both sea-island and upland cotton were sequenced in 2015, paving the way for the use of tools such as CRISPR/Cas9 in genetic improvement programs [20–22]. Recent studies using protoplast transient transformation or transgenic plants through stable transformation have demonstrated that CRISPR/Cas9 can be used for gene editing in cotton [23–28]. However, despite the great potential shown by these reports, cotton is a specially difficult crop with many obstacles that must be overcome before large scale genetic improvement programs can be implemented through gene editing. Upland cotton (*Gossypium hirsutum*) is a heterotetraploid and targeted mutagenesis is considered to be difficult as many genes are duplicated in this complex genome [20, 21]. In addition, CRISPR/Cas9 applications are hindered by the labor-intensive and lengthy transformation protocols. Cotton stable transformation is usually performed using *Agrobacterium tumefaciens* taking between 8 to 12 months to generate T_0_ plants [23, 29]. Maximizing CRISPR/Cas9-mediated mutation efficiency is critical in order to reduce workload and facilitate genome editing approaches in cotton.

Producing highly efficient and abundant sgRNA transcripts *in planta* is crucial for genome editing. Previously, we described a transient transformation system to rapidly validate the efficiency of sgRNAs [23]. In this study, we provide an additional alternative method to evaluate the efficiency of target sequences in CRISPR/Cas9-mediated target mutagenesis. Furthermore, we optimized the cotton CRISPR/Cas9 system by enhancing sgRNA expression using an endogenous *U6* promoter. The *GhU6.3* promoter produced higher sgRNA transcript levels than the *Arabidopsis AtU6*-*29*, leading to a 4–6 times increase in genome editing efficiency when used in CRISPR/Cas9 cassettes. Our work provides a faster and more efficient pipeline for the use of genome editing in cotton basic and applied research.

## Methods

### Plant materials and growth conditions

*Gossypium hirsutum* L. variety ‘TM-1’ seeds were imbibed in deionized water for 3 h then incubated at 28 °C for germination. The germinated seeds were transferred into soil and grown at 25/28 °C (night/day) and 16 h/8 h light/dark cycle. Seedlings were grown for 10 days before being used for *Agrobacterium* transformation [30]. Tobacco plants were grown in a greenhouse (25 °C at 16 h/8 h light/dark cycle) for 3 weeks before leaves were used for transformation [31].

### Promoter sequences analysis and vector construction

The *U6* snRNA (small nuclear RNA) sequences from *Arabidopsis* were used to identify the cotton *U6* gene using the genome of ‘TM-1’ as reference (http://mascotton.njau.edu.cn/info/1054/1118.htm). 1 kb fragments upstream of the predicted cotton *U6* genes were isolated and cloned for sequencing. Predicted promoter sequences were sub-cloned into pGWB433 by Gateway cloning (Invitrogen, USA). The *AtU6*-*29* promoter was cloned in to pGWB433 as positive control.

To construct the non-functional GUS vector, a 23 bp fragment that targets sgRNA-PDS [23] was inserted behind the *GUS* start codon. The modified *GUS* gene was called *fsGUS*, and cloned into pK2GW7.0 [31].

CRISPR/Cas9 vector construction was performed as previously described [23, 32]. Briefly, the *U6* promoter and sgRNA scaffold were integrated by PCR and then ligated into the CRISPR/Cas9 expression cassette by Golden Gate cloning (NEB, USA).

### Transient transformation in tobacco and cotton

All vectors were introduced into *A. tumefaciens* GV3101 for transient transformation, and GV3101 strains carrying the vector were grown in selection media at 28 °C. *Agrobacterium* cells were collected by centrifugation and suspended in infiltration medium [10 mM magnesium chloride, 10 mM 2-(N-morpholine) ethyl sulfonic acid, and 200 μm acetosyringone]. After incubating at room temperature for 3 h, the *Agrobacterium* suspension was infiltrated into tobacco leaves or cotton cotyledons. The experiments were repeated at least three times with more than 8 leaves per experiment [23, 33, 34].

### GUS staining and enzyme activity determination

GUS staining was performed by incubating infiltrated plant leaves in GUS staining solution for 10 h at 37 °C. Leaves were then incubated in 75% ethanol to remove chlorophyll. Stained samples were analyzed using a Leica microscope (USA) and GUS activity was determined as described in previous study [35].

### RT-PCR and qPCR analysis

Total RNA was isolated from cotton cotyledons 2 days after infiltration using the Aidlab RNA extraction kit (Aidlab Biotechnologies, China). First strand cDNA was synthesized from 1 μg of total RNA using the M-MLV reverse transcript system (Promega, USA). RT-PCR was performed at 95 °C for 3 min followed by 28–35 cycles of amplification (95 °C for 20 s, 55–60 °C for 20 s and 72 °C for 20 s). qRT-PCR was performed on an ABI 7500 Real Time PCR system (Applied Biosystems, USA) with SYBR green (Bio-Rad, USA). Relative gene expression levels were calculated using the 2^−∆∆Ct^ method with the cotton *Ubiquitin 7* gene (*UB7*) as the reference gene [33, 34]. Primers used for PCR amplification are listed in Additional file 1.

## Results

### Identification of *U6* promoters in upland cotton

**Fig. 1:**
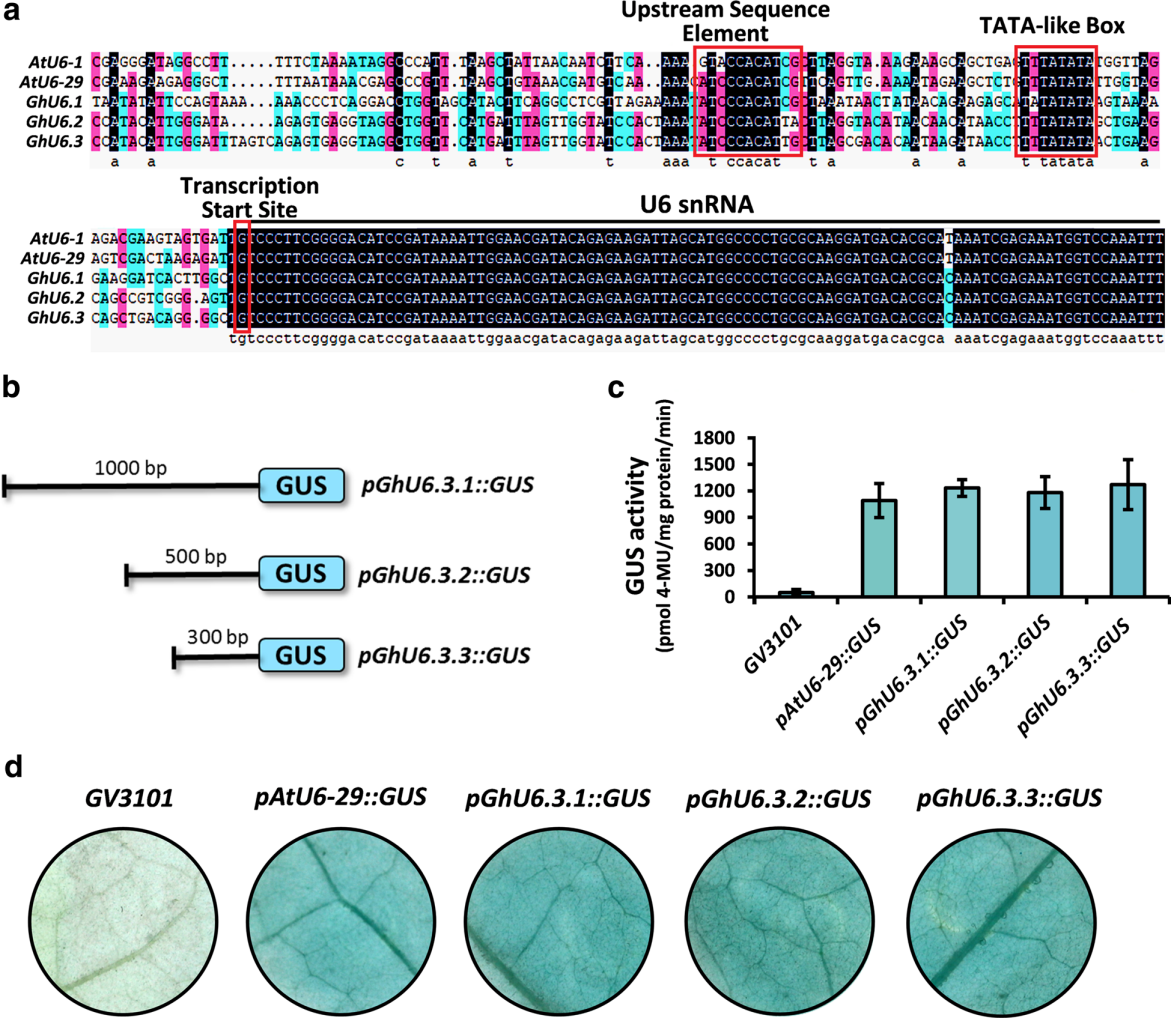
Identification and validation of cotton *U6* promoters. **a** Multiple alignments of cotton and *Arabidopsis U6* gene and promoter sequences. Black line denotes the *U6* snRNA transcript. USE (upstream sequence element), TATA-like Box and the transcription start site are labeled with red boxes. **b** Schematic representation of GUS expression constructs using different *GhU6.3* promoter fragments. **c** GUS activity levels and **d** GUS staining in tobacco leaves infiltrated with *Agrobacterium* carrying different promoter constructs. The *error bars* indicate the standard deviation estimated from the eight replicates

*Arabidopsis thaliana U6* promoters are commonly used in dicot CRISPR/Cas9 cassettes to drive the expression of sgRNAs as they are thought to achieve high expression levels. We have successfully used *AtU6*-*29* to mutagenize the *Cloroplastos alterados 1* (*CLA1*) gene in transgenic *G. hirsutum* plants [23]. In an effort to optimize the CRISPR /Cas9 system in *G. hirsutum*, we performed BLAST searches of the cotton genome using *AtU6*-*29* as query and identified three most homologous sequences showing high homology which were named *GhU6.1*, *GhU6.2*, and *GhU6.3* (Fig. 1a). Although the *U6* snRNA transcript sequences in cotton and *Arabidopsis* were almost identical, the promoter regions were very divergent except for the two motifs required for transcription, the upstream sequence element (USE) and the TATA-like Box (Fig. 1a). Among the *G. hirsutum* promoter sequences, *GhU6.2*, and *GhU6.3* showed strong conservation in the first 467 bp upstream from the start of transcription while *GhU6.1* was quite divergent (Additional file 2). The 1 kb sequences upstream of the transcription initiation site of three promoters were cloned into the pGWB433 vector upstream of the β-glucuronidase (*GUS*) reporter gene. Transient transformation experiments were performed in tobacco leaves by *Agrobacterium* infiltration using all promoter constructs. All the three promoters can promote *GUS* expression, and the *GhU6.3* promoter shows the most stable and strong promoting ability than *GhU6.1* and *GhU6.2* promoter (Additional file 3). Therefore, the *GhU6.3* promoter was chosen for further study (noted as *proGhU6.3.1*) and two genomic fragments of 500 bp and 300 bp (*proGhU6.3.2*, *proGhU6.3.3*) upstream of the transcription initiation site were cloned to build the vectors to drive *GUS* expression (Fig. 1b). An additional construct containing the *Arabidopsis AtU6*-*29* promoter was also prepared for comparison purposes. GUS staining and quantitative GUS activity assays showed similar staining intensities and GUS activity values for *pGhU6.3.1::GUS*, *pGhU6.3.2::GUS*, *pGhU6.3.3::GUS* and *pAtU6*-*29::GUS* (Fig. 1c, d). We therefore used the shortest fragment, *ProGhU6.3.3* (300 bp), to test its efficiency for CRISPR/Cas9-mediated gene editing in cotton.

### The endogenous *GhU6.3.3* promoter drives higher sgRNA expression levels than the *Arabidopsis AtU6*-*29* promoter resulting in higher CRISPR/Cas9-mediated mutation efficiency in cotton

**Fig. 2:**
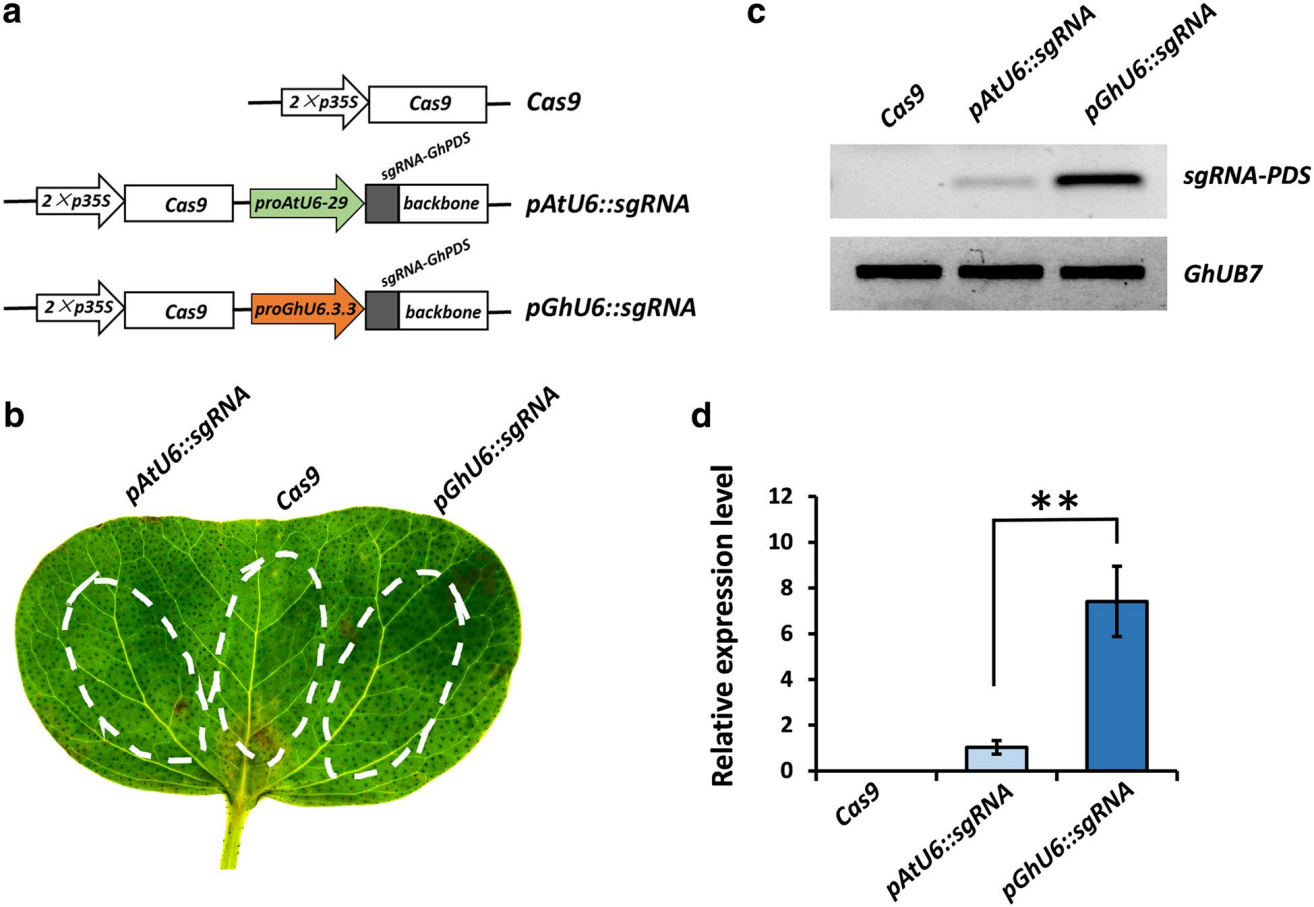
The *GhU6.3.3* promoter drives strong sgRNA expression in cotton. **a** Diagram of CRISPR/Cas9 expression vectors: *Cas9*, vector lacking sgRNA cassette used as negative control; *pAtU6::sgRNA*, CRISPR/Cas9 construct with sgRNA-PDS driven by *proAtU6*-*29*; *pGhU6::sgRNA*, CRISPR/Cas9 construct with sgRNA-PDS driven by *proGhU6.3.3*. **b** Schematic diagram of *Agrobacterium*-mediated transient expression in cotton cotyledon with different constructs. **c** sgRNA expression levels determined by RT-PCR and **d** qPCR (n > 8, **P < 0.01, *t*-test)

To compare the levels of sgRNA expression achieved by the *proGhU6.3.3* and *proAtU6*-*29* in cotton, we constructed two CRISPR vectors using each promoter to drive a previously designed sgRNA targeting the *Phytoene desaturase* (*PDS*) gene [23] while an empty vector (*Cas9*) was used as negative control (Fig. 2a). All constructs were introduced into *A. tumefaciens* GV3101 and transient expression experiments performed in cotton cotyledons by infiltrating each of the constructs in a different region of the same cotyledon (Fig. 2b). Cotyledons were harvested 48 h after infiltration and the levels of sgRNA-PDS transcripts quantified by RT-PCR and qRT-PCR. Our results showed that *proGhU6.3.3* generated 6–7 times higher levels of sgRNA-PDS expression than *proAtU6*-*29*, suggesting that it could be a better choice for CRISPR/Cas9 applications in upland cotton (Fig. 2c, d).

**Fig. 3:**
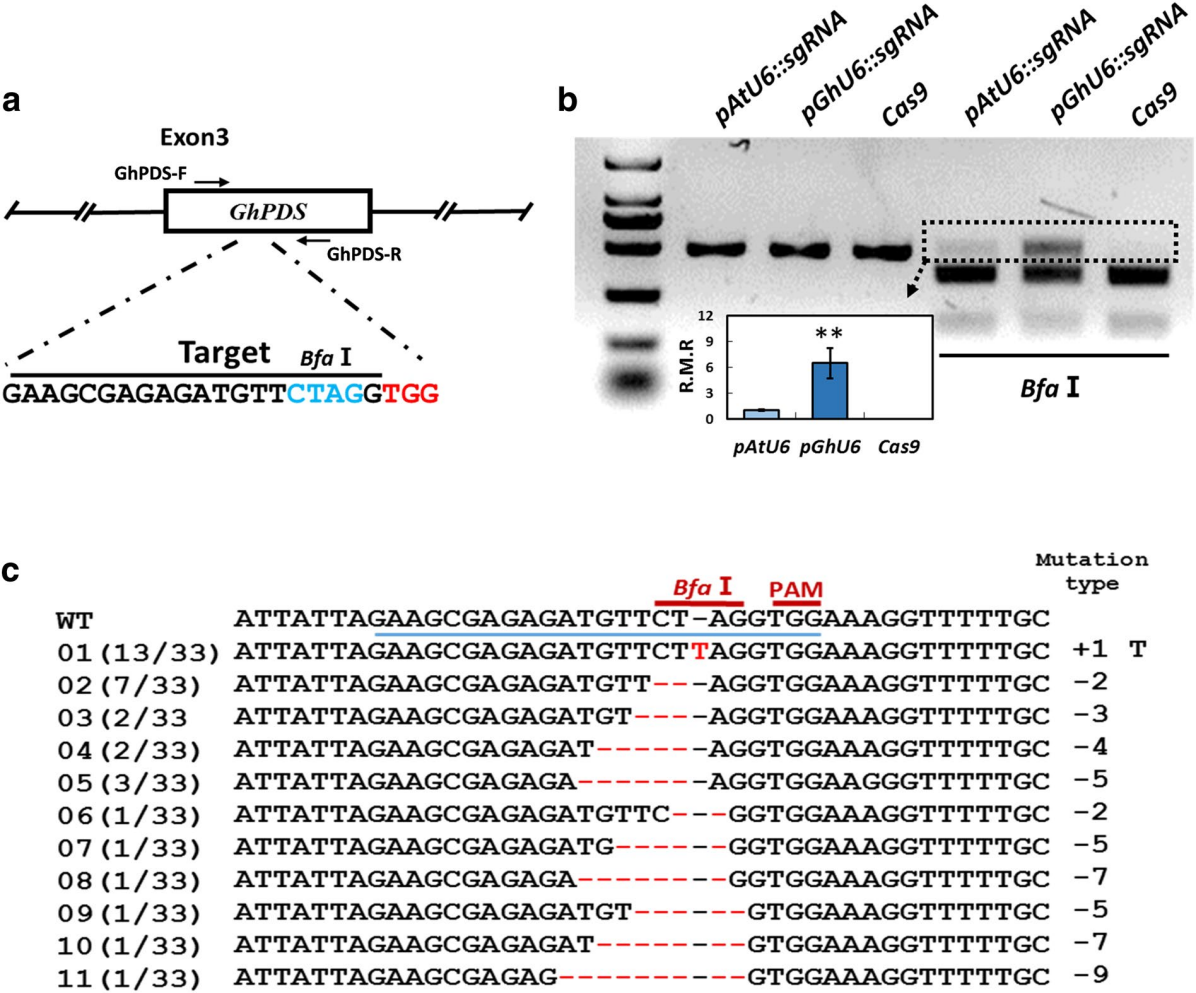
CRISPR/Cas9-induced targeted mutagenesis of *GhPDS* in cotton. **a** Position of the sgRNA-GhPDS target site in the *GhPDS* gene. GhPDS-F and GhPDS-R indicate primers used to amplify the target fragment. **b** Detection of CRISPR/Cas9 induced sgRNA-GhPDS mutations. Gel electrophoresis analysis of PCR amplicons of target fragments from *Agrobacterium* transient expression experiments using different CRISPR/Cas9 cassettes. 1–3: undigested PCR products; 4–6: PCR products digested with *Bfa*I. The insert (column-value histogram) shows the relative mutation rates (n > 8, **P < 0.01, *t*-test). **c** Sequencing of mutated PCR products. The sgRNA target sequence is underlined in blue. Deletions are shown as red dashes, and insertions are denoted with red letters. The frequency of each mutation is shown on the left and the mutation types on the right

To determine whether the increased sgRNA expression levels driven by the *GhU6.3.3* promoter resulted in improved genome editing efficiency in cotton, we performed transient expression experiments with the binary vectors described above (Fig. 2a). The target sequence for the sgRNA contains a *Bfa*I restriction site adjacent to the protospacer adjacent motif (PAM), thus CRISPR/Cas9-mediated genome-editing events were expected to alter the *BfaI* recognition sequence (Fig. 3a). Cotyledons were infiltrated with *Agrobacterium* containing each of the three CRISPR/Cas9 cassettes, *pGhU6::sgRNA, pAtU6::sgRNA* and *Cas9* (Fig. 2a), and tissue collected 3 days after infiltration. Genomic DNA was purified and a 486 bp genomic fragment containing the target sequence in the *PDS* gene amplified by PCR (Fig. 3b, lanes 1–3). Digestion of the amplicon with *Bfa*I should yield two fragments of 340 bp and 146 bp respectively in WT sequences while genome editing events modifying the *Bfa*I restriction site should result in the appearance of an uncut product. Electrophoretic analysis of the *Bfa*I digested amplicons showed the existence of uncut products in the *proGhU6.3.3* or *proAtU6*-*29* samples (*pAtU6::sgRNA* and *pGhU6::sgRNA*, Fig. 3b, lane 4, 5), but not in the control containing a vector lacking sgRNA (Fig. 3b, lane 6). Densitometry analysis showed that the intensity of the uncut band in amplicons from *pGhU6::sgRNA* samples was approximately 4–6 times stronger than those obtained in *pAtU6::sgRNA* samples (Fig. 3b).

The uncut bands from samples transfected with *pGhU6::sgRNA* and *pAtU6::sgRNA* were purified from the gel and cloned into a plasmid vector to investigate the nature of the editing events in the *GhPDS* target site. Sequencing analysis of multiple colonies showed that the types and proportion of mutations were similar in samples transfected with *pGhU6::sgRNA* and *pAtU6::sgRNA* with 61% of clones harboring small deletions (Fig. 3c and Additional file 4).

**Fig. 4:**
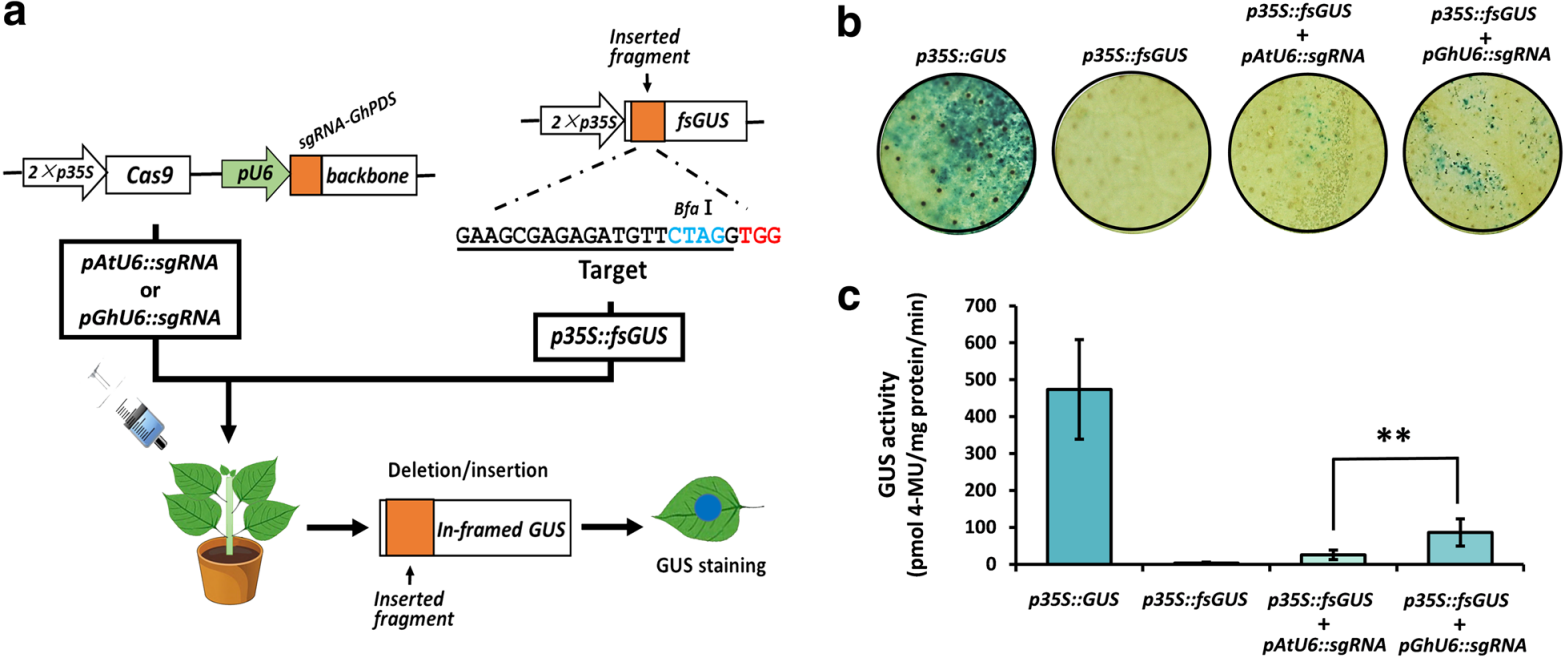
Detection of CRISPR/Cas9-mediated mutation efficiency using the frame-shift GUS (fsGUS) system. **a** Diagram of the fsGUS reporting system. The 23 bp target sequence of sgRNA-PDS was inserted after the *GUS* start codon to generate fsGUS (*p35S::fsGUS*). CRISPR/Cas9 constructs (*pAtU6::sgRNA* and *pGhU6::sgRNA*) were co-expressed with *p35S::fsGUS* in cotton cotyledons, the *p35S::GUS* was used as positive control. **b** GUS staining of cotton cotyledons co-transfected with fsGUS and CRISPR/Cas9 constructs. **c** GUS activity assays of cotton cotyledons co-transfected with fsGUS and CRISPR/Cas9 constructs (n > 8, **P < 0.01, *t*-test)

To confirm our results, we used the frame-shift GUS (fsGUS) reporter system [31] to further compare the efficiency of CRISPR/Cas9 cassettes containing either *proGhU6.3.3* or *proAtU6*-*29* to drive sgRNA expression. In this system, a 23 bp sequence including the sgRNA-PDS target was inserted into the *GUS* coding sequence, causing a frame shift and avoiding the production of functional GUS protein. CRISPR/Cas9-mediated gene editing typically generates a variety of mutations at the target site, some of which will correct the frame shift in fsGUS, resulting in a functional GUS protein that can be detected by staining and enzymatic activity assays (Fig. 4a). As shown in Fig. 4b, no GUS expression was observed in cotton cotyledons after infiltration with *Agrobacterium* harboring the *p35S::fsGUS* construct only. Cotyledons co-infiltrated with *p35S::fsGUS* and CRISPR/Cas9 constructs containing either *pAtU6::sgRNA* or *pGhU6::sgRNA* showed spots with blue coloration, suggesting the successful generation of mutations in *fsGUS*. In agreement with our previous results, co-infiltration with *p35S::fsGUS* and *pGhU6::sgRNA* produced a larger number of colored spots and a stronger color intensity than co-infiltration with *p35S::fsGUS* and *pAtU6::sgRNA* (Fig. 4b). Visual observations were complemented with GUS activity measures showing 4 times higher GUS activity values in the *pGhU6::sgRNA* samples (Fig. 4c).

## Discussion

Cotton accounts for 40% of the international fiber market making it irreplaceable in the world economy. Traditional breeding has failed to keep up with the demand for yield and quality improvement gradually making molecular breeding the method of choice for cotton as it can shorten the breeding cycle while maintaining high-quality traits. The sequencing of the diploid and allotetraploid cotton genomes, combined with recent transcriptomics and proteomics work [20–22, 36–40], have provided an invaluable resource for genetic studies and the development of innovative biotechnological approaches for cotton improvement. The CRISPR/Cas9 system allows the production of precise targeted mutations in the genome and can generate transgene-free mutants, potentially avoiding costly regulatory requirements associated with genetically manipulated crops [4, 6–8, 10, 16]. Although CRISPR/Cas9 has now been used in many crop species, its application in cotton is severely hampered by the long and technically challenging genetic transformation process, making it imperative to maximize its efficiency.

With a few notable exceptions, genetic transformation is a time consuming and labor intensive process emphasizing the need to optimize CRISPR/Cas9-mediated mutagenesis efficiency. The two core elements of the CRISPR/Cas9 system are the Cas9 nuclease and the associated sgRNA. Previous studies have shown that gene editing activity is strongly reliant on achieving strong expression of both elements and thus the choice of promoters is essential for the overall mutagenesis efficiency [7–10, 17]. Strong constitutive RNA polymerase II promoters such as the cauliflower mosaic virus 35S (CaMV 35S) and the ubiquitin promoters are typically used for the control of Cas9 expression in stably transformed plants obtained using tissue culture-based protocols [10, 32]. In contrast, the CaMV 35S promoter has proven to be sub-optimal for *Arabidopsis* transformed using the floral dipping method with germ line specific and cell division specific promoters proving vastly superior [41]. Improvements have also been achieved by optimizing Cas9 codon usage for the species of interest in order to enhance translation efficiency [42]. Transcription of the sgRNA molecule has been usually controlled by RNA polymerase III dependent promoters such as the *U6* promoters. *U6* promoters have a number of advantages such as their precise start of transcription and the tight control over the length of the transcript. In addition, *U6* promoters are usually active in multiple species with the *Arabidopsis U6* promoter able to direct strong transcription in tobacco, tomato, poplar and other dicotyledonous species [11, 12, 32]. Nevertheless, there are limitations to the ‘universal’ nature of *U6* promoters as the *Arabidopsis U6* promoter was inefficient in wheat and rice [43]. There is also evidence suggesting that the use of endogenous *U6* promoters can improve the efficiency of CRISPR/Cas9 systems such as in soybean, where the *GmU6*-*10* promoter produced 2–6 times higher mutation efficiency than the *Arabidopsis U6*-*26* [17]. Finally, not all target sequences are equal, with different targets producing different mutation efficiencies perhaps due to secondary structure factors caused by the GC content [44].

In crops such as cotton, with lengthy and labor intensive transformation protocols it is essential to select the best possible CRISPR/Cas9 system before attempting stable transformation. We have previously developed a transient transformation protocol combined with restriction enzyme digestion of the targeted genomic loci to validate and assess the functionality of different sgRNAs in cotton [23]. Here, we provide a second independent method to further validate target sites using the *fsGUS* system. With this system, we validated different CRISPR/Cas9 constructs in 3 days using simple experimental techniques such as *Agrobacterium* infiltration combined with GUS staining and activity assays.

Achieving high sgRNA expression is essential for effective mutagenesis. We show here that a 300 bp *GhU6.3* promoter fragment is enough to drive consistent gene expression in tobacco with similar expression levels than the *Arabidopsis AtU6*-*29*. In contrast, when both promoters were tested in cotton, we found that the sgRNA transcript levels driven by the endogenous *GhU6.3* were 6–7 times higher than those driven by the *Arabidopsis AtU6*-*29*. Our results are consistent with previous reports suggesting that endogenous *U6* promoters produce higher expression levels than non-endogenous promoters [17]. The increased expression levels were reflected on higher mutagenesis efficiencies with *GhU6.3* resulting in 4–6 times higher mutagenesis rates than *AtU6*-*26* measured with two independent methods, in agreement with pervious observations in other species [9, 17, 24]. As expected, the types mutation produced by both of promoters were similar. Even though we have previously used the *Arabidopsis AtU6*-*29* promoter to successfully generate gene editing in cotton [23], the increased efficiency provided by *GhU6.3* will be extremely useful to reduce the number of transgenic lines needed to ensure the generation of mutants, especially in the case of the heterotetraploid *G. hirsutum* or if multiple genes are simultaneously targeted.

## Conclusion

In summary, we provide a fast and effective method to validate sgRNA mutagenesis efficiency in cotton using CRISPR/Cas9 and transient expression methods. We also provide an improved CRISPR/Cas9 cassette using an endogenous *U6* promoter to drive sgRNA expression that generates improved mutagenesis efficiency over the existing one. Generating stable transformation of cotton is time-consuming and labor-intensive and thus the improvements should result in important savings for research groups using CRISPR/Cas9 in cotton.

## Additional files

**Additional file 1.** List of primers used in this study.

**Additional file 2.** Multiple alignments of *proGhU6.1*, *proGhU6.2* and *proGhU6.3* sequences. The USE and TATA-box were boxed, the transcriptional start site was marked with star.

**Additional file 3.** The GUS staining in tobacco leaves infiltrated with *Agrobacterium* carrying different promoter constructs.

**Additional file 4.** Sequencing of mutated PCR products generated by *pAtU6::sgRNA*. The sgRNA target sequence is underlined in black. Deletions are shown as red dashes, and insertions are denoted with red letters. The frequency of each mutation is shown on the left and the mutation types on the right.
