# Supplementary figures and images for: Optimization of CRISPR/Cas9 genome editing in cotton by improved sgRNA expression
Source: Plant Methods. 2018 Oct 3;14:85. doi: 10.1186/s13007-018-0353-0 (PMC6169012; doi:10.1186/s13007-018-0353-0)

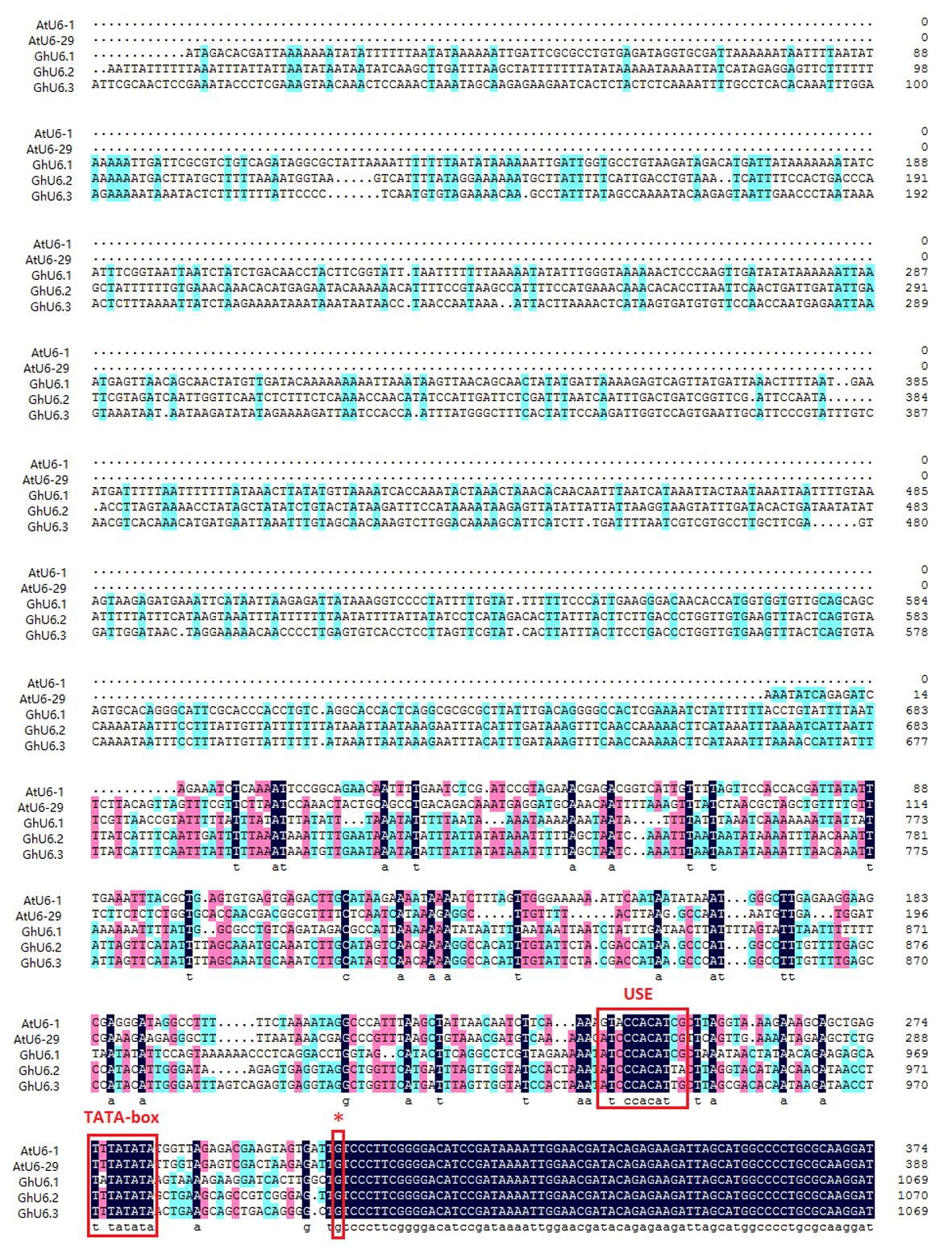

Supplement: Supplementary file 2 — Additional file 2. Multiple alignments of proGhU6.1, proGhU6.2 and proGhU6.3 sequences. The USE and TATA-box were boxed, the transcriptional start site was marked with star. [file 13007_2018_353_MOESM2_ESM.png]

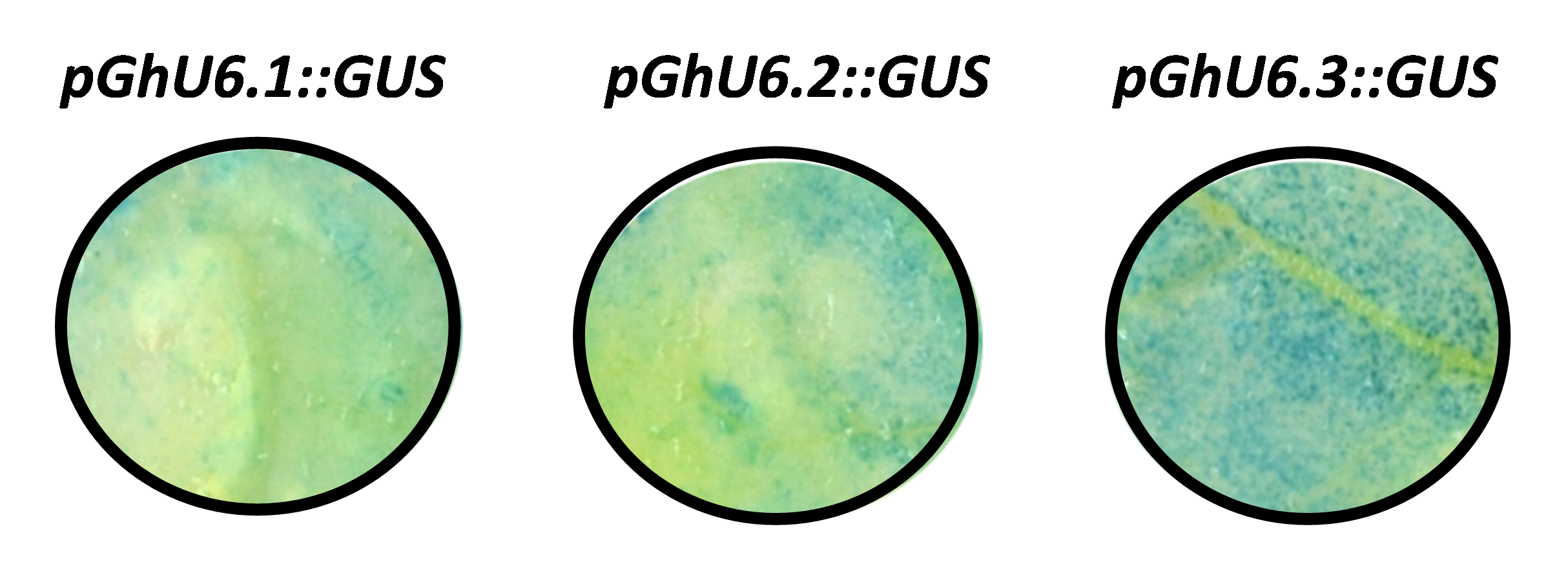

Supplement: Supplementary file 3 — Additional file 3. The GUS staining in tobacco leaves infiltrated with Agrobacterium carrying different promoter constructs. [file 13007_2018_353_MOESM3_ESM.png]

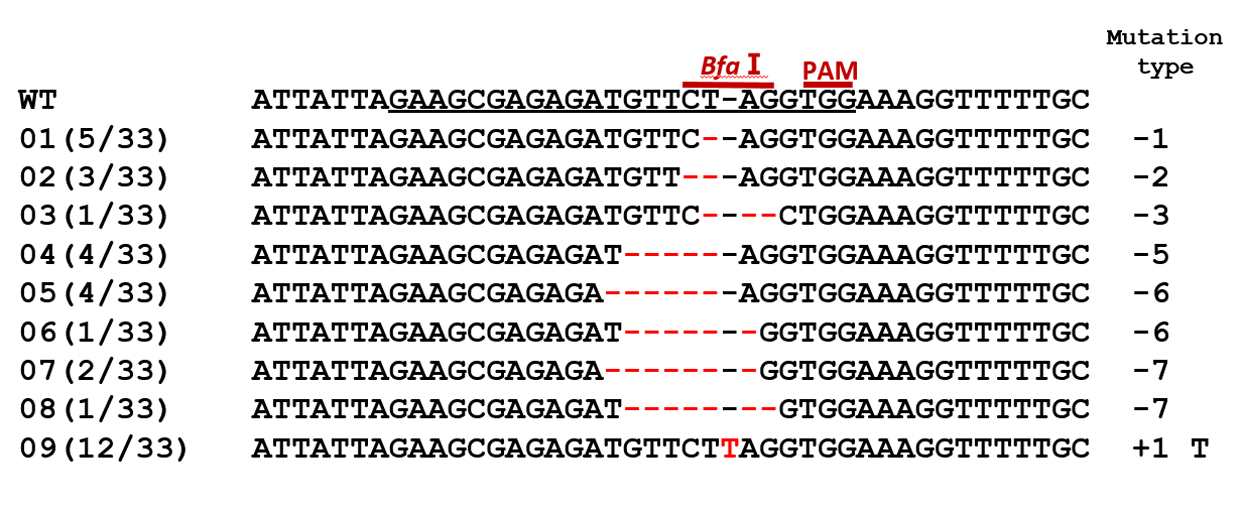

Supplement: Supplementary file 4 — Additional file 4. Sequencing of mutated PCR products generated by pAtU6::sgRNA. The sgRNA target sequence is underlined in black. Deletions are shown as red dashes, and insertions are denoted with red letters. The frequency of each mutation is shown on the left and the mutation types on the right. [file 13007_2018_353_MOESM4_ESM.png]
